# How international research consortia can strengthen organisations’ research systems and promote a conducive environment and culture

**DOI:** 10.1136/bmjgh-2022-011419

**Published:** 2023-04-07

**Authors:** Justin Pulford, Taghreed El Hajj, Tara Tancred, Yan Ding, Susie Crossman, Lorelei Silvester, Martina Savio, Natasha Bevan, Nadia Tagoe, Imelda Bates

**Affiliations:** Liverpool School of Tropical Medicine, Liverpool, UK

**Keywords:** Review

## Abstract

Research systems and cultures have been criticised for their detrimental effect on members’ mental health and well-being. Many international research programmes operate through research consortia that have the resources to make a substantial contribution to improving the research environment in their member organisations. This paper collates real-life examples from several large international consortia-based research programmes about how they strengthened organisations’ research capacity. The consortia primarily involved academic partners from the UK and/or sub-Saharan Africa and covered research topics including health, natural sciences, conservation agriculture and vector control. They were partly or wholly funded by UK agencies including the Wellcome, Foreign and Commonwealth Development Office, UK Research and Innovation Fund, and the Medical Research Council and they operated for 2–10 years between 2012 and 2022.

Consortia’s size and ability to access and share resources among their member organisations according to need meant they were uniquely placed to target actions to address weaknesses in member organisations’ research capacity, to widen networks and collaborations, and to build in sustainability of capacity gains. Consortia’s actions covered: (a) individuals’ knowledge and skills; (b) capacity strengthening ethos; (c) organisations’ visibility and prestige; and (d) inclusive and responsive management practices. Evidence about these actions formed the basis of recommendations for funders and leaders of consortium-based programmes about how they could make more effective use of consortia’s resources to enhance organisations’ research systems, environments and cultures.

Key lessons were that training should cover management and research leadership and should be offered beyond consortium members, including to research support staff such as technicians and managers. Consortia often tackle complex problems requiring multidisciplinary inputs, but overcoming disciplinary boundaries—and making everyone feel valued and respected—takes time and skill on the part of consortium leaders. Consortia need clear guidance from funders about their commitment to strengthening research capacity. Without this, consortia leaders may continue to prioritise research outputs over creating and embedding sustainable improvements in their organisations’ research systems.

**SUMMARY BOX**

- Large international research programmes often operate through research consortia; these consortia have access to experts, equipment and other resources that could substantially improve the research environment in their member organisations.
- A lack of evidence and scarce examples of practical actions mean that consortia, and their funders, are not optimising opportunities to improve the research environment in their member organisations.
- Principles of equity, inclusiveness and transparency should underpin consortia’s approach to all aspects of research capacity strengthening.
- Actions that consortia take to strengthen organisations’ research systems, environments and culture are wide-ranging and can involve enhancing individuals’ knowledge and skills, instilling a capacity strengthening ethos, raising organisations’ visibility and prestige, and creating inclusive and responsive management practices.
- Consortia are uniquely able to identify weaknesses in their member organisations and, because of their access to resources and networks, to also address these during their project’s lifetime.
- Consortia can facilitate new collaborations—including among low/middle-income country partners—not just for conducting research, but also for sharing expertise to sustainably strengthen organisations’ research management and support systems.
- Funders of research consortia need to make it clear in their guidance, and in their processes for selecting and evaluating projects, that strengthening research systems and cultures is a priority; this will help consortium leaders in their activity planning, budgeting, trouble-shooting and decision-making.
- Demand for research should be increased to create much more evidence about how to evaluate the research capacity strengthening components of consortia-based programmes and on better, validated tools and indicators to do this; these evaluation processes should be constructive for funders and grantees, and not overly burdensome.
- Consortia should make more use of evidence and have a better understanding about how to harness their power and resources to achieve a substantial positive shift in organisations’ research systems and environment.
- Research environments and cultures that are efficient and supportive should be the goal so that all disciplines and everyone’s contributions are valued; this will motivate and retain a vibrant workforce and enhance organisations’ ability to respond to national priority needs.

## Introduction

Many large international research programmes operate through research consortia. These consortia comprise groups of scientists from multiple organisations cooperating together on a specific research topic. Consortia are able to address scientific questions that are complex and need pooled resources and/or expertise to solve. Some international consortia may have an additional objective to strengthen the research capacity of their partner organisations in low/middle-income countries (LMICs)—this may be a primary objective or secondary to the research. It is these dual-purpose consortia involving LMICs, where the partner organisations may have widely differing capabilities, that are the focus of this paper.

In the past, research consortia tasked with enhancing research capacity often concentrated on training scientists and researchers.^1 2^ This focus left organisational systems underequipped to manage and support the delivery of research^3 4^ because to be resilient and to sustainably generate high-quality research, organisations do not only need a skilled workforce, but effective research systems. These systems should promote integrity, a ‘creative, inclusive and honest’ culture and an environment that motivates the research workforce and puts them at the centre.^5 6^

Research consortia, especially those with a remit to strengthen research capacity, are now increasingly being led by LMIC organisations. This helps to address power asymmetries and ensures responsiveness to national priorities.^7–9^ There is also more focus on strengthening organisational research systems. For example, the £56 million Developing Excellence in Leadership, Training and Science (DELTAS)-Africa phase two programme ‘strongly encouraged’ applicants with the ‘ability to build strong research cultures and environments’.^10^ However, feedback from >4000 researchers (including 24% from outside the UK) showed that many researchers are dissatisfied with their research environments and culture, partly because the commonly used metrics of research success (ie, publications and grant income) drive ‘unhealthy competition, bullying and harassment’ and negatively affect mental health.^11^

## The role of consortia in reforming and strengthening organisations’ research systems and cultures: real-life learning

Research consortia are able to influence improvements in research systems and cultures in their member organisations, but in practice they and their funders are not maximising this influence. They are hampered by a lack of evidence and are generally not incentivised to invest in research systems unless strongly encouraged by their organisations’ leaders or mandated by the funders. Even if funders do require them to strengthen organisational systems, cultures and environments, the resources provided to do this are often relatively small compared with the funds available for their primary research. The importance of taking a systems approach has been widely recognised because capacity development is ineffective if targeted at only one level—it needs to extend beyond individuals to organisations and the (inter)national level. Not taking a systems approach means that capacity development activities are fragmented, local institutions are not able to train and retain their researchers,^1^ and there is a lack of national bodies able to coordinate research and use findings for policy and practice.^7^

Although some tools to help organisations identify strengths and gaps in their research systems have been published,^12 13^ there is insufficient evidence about how to strengthen research systems systematically and effectively, and how to measure any changes in outcomes and impact.^14^ In particular, evidence about how this strengthening can be achieved *by research consortia* is scarce.^15–17^ Unless the outputs and outcomes of research system strengthening can be measured, consortia leaders and funders may find it difficult to allocate resources to these activities since they both rely on project evaluation indicators to demonstrate performance.^18^

For over a decade, our Centre for Capacity Research (CCR) has been producing evidence about how to improve the efficiency, effectiveness and inclusiveness of organisations’ research systems—especially within international research consortia primarily involving partners in the UK and sub-Saharan Africa. Our CCR researchers are multidisciplinary and, uniquely, have been embedded within large multinational research programmes at the interface between the programme management team and the consortia. They generate evidence that helps to improve programmes’ effectiveness in strengthening research capacity within the programmes’ lifetime while also contributing to the development of a critical mass of global knowledge and expertise.

The purpose of this paper is to collate practical examples of how large multinational research consortia influenced and strengthened member organisations’ research systems. Illustrative examples of actions taken, which were drawn from a range of programmes, are provided in italicised boxes. We present learning and recommendations for consortium leaders and their members, and for their funders and programme managers, about practical actions they can take to contribute more effectively to positive changes in research environments and culture among their member organisations. Our recommendations are based on our reflections about evidence generated from many large consortia-based research programmes which focused on research topics including health, natural sciences, conservation agriculture and vector control. These programmes all had a mandate from the funders to strengthen research capacity in their LMIC partner organisations.

The programmes and their participating consortia had all been selected by the funders following open competitions. The consortia all involved organisations (mostly academic) in LMICs (predominantly sub-Saharan Africa) with variable levels of management and research capacity. Further details about the programmes from which evidence was generated are provided in box 1. This evidence was collected using multiple methods (eg, surveys, observations, interviews, document reviews) and from the perspectives of different cadres in the research workforce (eg, researchers, managers, directors, funders, technicians, evaluators).

Box 1 **Brief description of illustrative programmes from which learning and recommendations were derived**

- **Developing Excellence in Leadership, Training and Science (DELTAS)-Africa, phase one (2015–2022**).^48^ A US$100 million health research capacity strengthening programme implemented by the Alliance for Accelerating Excellence in Science in Africa with support from the Wellcome Trust and the UK’s Foreign, Commonwealth and Development Office (FCDO). DELTAS-Africa funded 11 African-led research consortia to implement cutting-edge collaborative health research and training programmes spanning 54 organisations from across Africa. Centre for Capacity Research’s research focused on factors that promote and hinder equitable PhD programmes, researchers’ capability in knowledge exchange and the processes involved in the management of consortia.
- **International Multidisciplinary Programme to Address Lung Health and Tuberculosis in Africa (IMPALA 2017–2021**).^49^ A £7 million 4.5-year programme, funded by the National Institute for Health Research, aimed to generate knowledge and implementable solutions concerning lung health and tuberculosis. Led by a Global North research institute, IMPALA had 22 international partner organisations from 13 countries, 10 in sub-Saharan Africa. IMPALA explicitly used multidisciplinary approaches and spanned biology to policy.
- **The African Capacity Building Initiative (ACBI 2012–2022**)^50^ is a £15 million pilot programme, which aims to strengthen the research and training capacity of higher education organisations and support the development of individual scientists in sub-Saharan Africa through UK–Africa research collaborations. Funded by FCDO in partnership with the Royal Society, ACBI supports 10 research consortia, each comprising one UK and three African organisations. Research within ACBI focuses on water and sanitation, renewable energy and soil-related science. The ACBI programme supports 38 PhD students from 26 African research organisations across 18 African countries.
- **Strengthening Capacity in Environmental Physics, Hydrology and Statistics for Conservation Agriculture Research (CEPHaS 2018-2021)**.^51^ A £5.1 million programme funded by the UK Research and Innovation Global Challenges Research Fund (GCRF) aims to build capacity in cross‐disciplinary research in soil physics, shallow geophysics, hydrogeology and environmental statistics in Malawi, Zambia and Zimbabwe. It undertakes learning‐centred demonstration trials to build capacity for cross-disciplinary research in soil physics, shallow geophysics, hydrogeology and environmental statistics to evaluate the impacts of conservation agriculture practices on the resilience of food production and water security under climate change.
- **Partnership for Increasing the Impact of Vector Control (PIIVeC 2018-2021)**^52^ is a £6.5 million programme funded by the UK Research and Innovation GCRF. It aims to stimulate the vector control research pipeline by investing in promising future leaders of vectorborne disease research, filling knowledge gaps and ensuring the sustainable use of evidence in decision-making at all levels. It brings together research institutes and national disease control programmes from Burkina Faso, Cameroon and Malawi, to undertake rigorous evaluation of the capacity needs in-country and develop evidence-based solutions.
- **A public health intervention development project (PHIND 2019-2021)**^53^ a £150 000 project funded through the UK Medical Research Council PHIND call. It was an early-phase study with partners in Ghana and Uganda to develop an intervention to understand the key barriers that health facilities and communities face in the prevention, management and treatment of postpartum haemorrhage. Key to the project was engagement of local stakeholders—community members, health facility staff, transfusing facility and blood bank staff, and staff from the Ministry of Health and national blood transfusion services.

Our authors were involved with these consortia for their whole duration either as researchers embedded in the programmes to explore aspects of research capacity strengthening or as research managers (MS, NB) who worked closely with the research teams. Ongoing qualitative and quantitative information on research systems was collected from across the programmes with additional in-depth investigations of specific aspects of research systems drawn from selected consortia. Our researchers synthesised the evidence and presented it back to consortia members for their feedback throughout the programme (eg, through presentations and seminars at annual meetings, reports and publications). More details about the programmes, the methods used to obtain information about research systems and associated publications can be found at CCR|Liverpool School of Tropical Medicine (lstmed.ac.uk). The researchers involved in each programme met regularly to reflect on and summarise findings and five (representing all of the programmes) came together at workshops in 2021–2022 to compare findings concerning research systems, culture and environment across all the programmes. Through these discussions, we identified examples of practical actions that consortia had used to strengthen organisations’ research systems and cultures and challenges they encountered. Four complementary groups of examples emerged, acting on different levels of the research system: (a) individuals’ knowledge and skills; (b) capacity strengthening ethos; (c) organisations’ visibility and prestige; and (d) inclusive and responsive management practices. Our recommendations are based on our findings and these examples.

## Individuals’ knowledge and skills

### Providing training for individuals that also explicitly benefits their organisations

Professional development opportunities were included in the budgets of all consortia’s projects and many consortia used these judiciously to simultaneously strengthen research systems. Within the programmes, each consortium developed its own approach to training. At the start of their projects, some consortia carried out training needs assessments primarily focusing on researchers, whereas others identified everyone from their members’ organisations who was funded by the consortium (including, for example, managers, laboratory and information technology (IT) technicians, finance officers and societal partners such as patients or community members) so they could provide equitable opportunities for professional development.

*International Multidisciplinary Programme to Address Lung Health and Tuberculosis in Africa (IMPALA), African Capacity Building Initiative (ACBI) and Partnership for Increasing the Impact of Vector Control (PIIVeC) consortia all involved multidisciplinary research. They used a baseline mapping of all those involved in a consortium from each organisation and developed a needs assessment for each person. Individualised training plans were tailored to individuals’ skills and knowledge gaps. Organisations’ leaders were involved in developing these training plans to make sure they aligned with the needs of their organisations.*

To help build a critical mass of expertise in organisations, or to strengthen organisations’ research support systems, consortia also often invited staff in their member organisations who were not funded through their programme, to training sessions.

*Consortia involved in the ACBI programme were encouraged to extend training opportunities beyond those directly funded through the programme. Individuals working in consortia’s organisations (eg, MSc and doctoral students, laboratory technicians and research professional services) were therefore invited to training and conferences provided by the consortia.*

Other consortia invited individuals from projects that were working on a similar research topic but who were not part of the consortium to events that they were hosting in their location. This added value to projects’ research capacity strengthening activities and also contributed to reducing their carbon footprint. Opportunities were provided for enhancing both technical and ‘soft’ skills (eg, leadership, management) through formal courses and often also through learning-by-doing. Consortia demonstrated flexibility so they could meet emerging training needs as these arose, and so they could better support new or different research cadres who may otherwise have been underserved in the original planning.

*Some consortia (eg, in DELTAS programme) purposefully recruited organisations’ existing academic staff into PhD or postdoctoral positions and developed research career pathways for them to ensure their enhanced skills were retained and used within their organisation at the end of their training.*

Several consortia involved organisations in non-English-speaking countries. They needed to include time and budget for translations to make sure that individuals from these organisations could fully engage in the programme.

*Ways in which consortia in PIIVeC, ACBI, IMPALA and DELTAS catered for participants from non-English-speaking countries included:*

- *Providing an English tutor and/or English language training to improve language skills for doctoral students, researchers and laboratory technicians.*
- *Budgeting for and translating training materials into, for example, French, Arabic and Amharic.*
- *Providing simultaneous translations during meetings.*
- *Organising immersion visits to English-speaking partner organisations for researchers and managers.*

### Valuing all contributions and disciplines

Most of the research undertaken by the consortia tackled complex problems and needed a multidisciplinary approach. Several consortium leaders therefore took active steps to demonstrate respect for, and promote mutual understanding of, the different disciplines of their member organisations recognising this helped to promote a vibrant and creative research environment within their consortium.

*In the IMPALA programme, time for individuals from different disciplines to come together was incorporated into project planning and included in project reporting. For example, meetings were organised to explicitly discuss the inter-relationships of different disciplines within the programme.*

Leaders were also aware that perceived or real hierarchies among different disciplines (eg, clinical trials were generally better funded, understood and respected than the social or implementation sciences) could lead to power imbalances and they made efforts to establish channels for listening to concerns from all consortium members.

*In IMPALA, training was provided on how to package findings for policymakers. In response to requests from consortium members, this was moved from the end to the start of the project so they could use this knowledge to design their data collection tools and analysis.*

*Bespoke training materials (videos, written guides) and online sessions (during the COVID-19 pandemic) were provided by the public health intervention development (PHIND) project to support data collection and analysis by research teams from different organisations working with communities. Study instruments were all co-designed, with live changes made to tools online during virtual meetings. This, and a new WhatsApp group to ensure timely responsiveness to study team queries and concerns, helped to engender equity, a team spirit and joint problem-solving, making everyone feel valued.*

Fostering multidisciplinary relationships and overcoming disciplinary boundaries took a lot of time and effort, especially at programme start-up, and was often underestimated. PhD students seemed to find it particularly difficult to engage with other disciplines, partly because they were on time-constrained programmes and were in the early stage of their career and therefore less independent in their research.

*All ACBI’s consortia members (principal investigators and co-investigators, PhD students, technicians, project coordinators) were involved in project management, stakeholder engagement and training, and fieldwork involving different research teams. These shared experiences promoted a team mentality, fostered understanding, respect and solidarity, and created alliances across disciplines and seniority, enabling a focus on common goals and preventing individual views from dominating.*

## Capacity strengthening ethos: sharing, encouraging and networking

Consortia were often able to enhance postgraduate training programmes in their partner organisations; for example, by providing mentors, supervisors or technical experts from within and beyond their own organisation for PhD students. In addition to creating new relationships across organisations, this motivated students and improved their performance and confidence, contributing to a more constructive research environment.

*Member organisations in ACBI and DELTAS programmes received visits and mentoring from another LMIC organisation to develop their financial systems and grant management in general and so they could manage funds in line with funders’ guidelines—this facilitated the transfer of funds and procurement of consumables and equipment and also the sourcing of their own external research funds.*

*Through the networks of organisations in PIIVeC and IMPALA, early career researchers were mentored to contribute to high-level national and international meetings (eg, technical working groups; United Nations General Assembly).*

Several consortia facilitated the sharing of equipment, training and expertise among their members (eg, on laboratory techniques or project financial management) and supported exchange visits (including between LMIC organisations) for all those involved in the consortium. In addition to providing technical skills and exposure to good practices concerning research cultures elsewhere, this inclusive approach clearly signalled that all members were valued and acknowledged.

Some consortia had budget allocations to support capacity strengthening for organisations’ priorities rather than for the specific needs of the research programme, for example, to develop guidelines or improve internet provision.

*Some organisations in the DELTAS programme received support from consortia to develop postgraduate supervision guidelines and contracts: others had funds allocated to improve internet bandwidth to enhance research activities. Other support for non-project-specific research capacity strengthening provided by PIIVeC included upgrading communal staff facilities.*

Occasionally, if consortia had more than one partner in the same country or city, there were opportunities to share office space or even jobs. Where common needs were identified across several consortia, access to central support was provided through the programme. The process of sharing resources among organisations helped to make the strengths of organisations explicit and also enhanced the technical capability and systems of weaker organisations within the consortia.

*IMPALA addressed weaknesses in their member organisations’ capacity by providing cross-organisational support and training for data management, statistics and research communication. Others (eg, ACBI and DELTAS) shared expertise and resources from better-capacitated organisations to help weaker organisations. This covered, for example, technical training, exchange of scientific expertise and knowledge, sharing equipment and other resources, joint publications, support with supervision and mentoring, on-site help with financial management and a project coordinator to reduce research leaders’ workload.*

Some consortia incorporated activities that would enable income generation, upgrading and expansion of their member organisations’ services, thereby fostering sustainability. This was particularly apparent in consortia that involved laboratory testing (eg, for water and soil analyses), because if these tests were externally certified to be of internationally recognised quality, they could market their services at commercial rates.

*With support from funders and grant managers, consortia in the ACBI programme developed an inventory of project equipment purchased by each consortium, and for each item a written sustainability plan was agreed with their African organisations to ensure continued use and maintenance of the equipment once the programme had finished. ACBI laboratory technicians requested, and received, training to help them achieve international accreditation (eg, on laboratory quality management systems) because this would make their organisations competitive and attractive to industry and research partners.*

Consortia leaders also had to overcome difficulties in personal relationships within and between organisations, for example, between: supervisors and PhD students; experienced and less experienced researchers; researchers with different disciplinary backgrounds; and researchers and other professional staff such as technicians and managers. To address these problems, consortia leaders took time to familiarise themselves with the cultural, social and political context of their member organisations. They provided opportunities for confidential discussions, shared policies for dealing with bullying and harassment, and made sure that everyone understood that they had a responsibility for safeguarding.^19 20^

*Interorganisational, independent monitoring of PhD student progress was established in several consortia (ACBI, DELTAS, IMPALA) along with additional supervision, mentoring and/or psychosocial support, which was available to all members of the research team.*

## Enhancing organisations’ visibility and prestige

Many consortia capitalised on their size and networks, and the visibility associated with a large research programme, to have greater influence on research uptake than would have been possible by each organisation acting alone. Organisations’ knowledge of the local context helped consortia make sure there was a demand for the research before starting, and ‘influencers’ within their organisations provided access to national decision-makers who could guide and use the research.

*Mechanisms that consortia used to make sure their organisations’ research met priority needs and to promote uptake of their findings included:*

- *Early consultations with research users so their priorities and feedback were incorporated into the research design (IMPALA*).
- *Involvement of patients and/or decision-makers in the research process itself (eg, as PhD students or consortium advisors, or as co-lead investigators) (IMPALA, PHIND).*
- *Involving national authorities and communities living in areas where the research was taking place in designing and implementing the research (ACBI*).
- *Having explicit cross-cutting activities on research uptake and policy engagement (IMPALA*).
- *Creation of a new sustainable, national technical working group involving researchers and policymakers (PIIVeC)*.

Some consortia employed specialists in policy engagement and research dissemination to promote impact—expertise that was often not available within individual organisations.

*DELTAS’s funders organised a training programme in Policy Engagement and Evidence Uptake for trainees across several consortia to promote research dissemination and enhance the visibility of their research.*

Organisations that have a reputation for impactful research are more likely to be sustainable, so several consortia adopted strategies (eg, ‘communities’, meetings) to maintain organisations’ enhanced networks once funding had ended.

*To promote long-term sustainability of research networks, the consortia:*

- *Set up e-platforms among researchers, managers and laboratory technicians (eg, WhatsApp groups) (PHIND*).
- *Held annual pan-consortium networking meetings or team retreats (ACBI, IMPALA).*
- *Constituted technical working groups across organisations (CePHAS*).
- *Arranged attendance at international conferences for networking and dissemination (IMPALA)*.

Some consortia fostered new interorganisational collaborations (eg, for proposal writing or training PhD students) involving activities beyond those of the original programme.

*Several DELTAS consortia came together into a coalition to jointly apply for funded programmes to enhance future capacity strengthening activities.*

*Examples of new collaborations that emerged from ACBI included:*

- *Joint publications, research grants and projects.*
- *Agreed sharing of laboratory equipment.*
- *A joint PhD programme enabling PhDs to be awarded in research areas where capacity was not available in an individual organisation. This was based on fee sharing and a partnership agreement that maintained separate quality manuals for the pathways in each organisation, with both organisations benefitting from sharing good practice.*
- *Establishing a joint research centre*.

## Engagement in inclusive and responsive consortium management practices

Managing the interface between the consortia research systems and those of their members’ organisations was a challenge for consortium leaders because they had to navigate multiple bureaucracies. They found ways to avoid setting up consortium-specific staff or systems in parallel to those in their partner organisations, aiming instead to build on existing systems as far as possible. They also introduced strategies to bridge the interface between the consortia’s systems and those of their member institutions, for example, by embedding consortia staff in member organisations and having equitable representation in consortia leadership teams.

*A DELTAS consortium established a management board made up of representatives from all member organisations and organisation-level management groups. The board was responsible for planning, implementing and reporting on the consortium’s activities within member organisations. In response to requests to distribute tasks and resources more equitably, the IMPALA programme introduced a rotational system among different discipline leads and the consortium directors for chairing consortium management meetings.*

Consortia leaders initiated early discussions with each of their member organisations to make sure they fully understood and were engaged with their research activities and objectives: this was particularly important for new members of the consortium and for those who had not been involved in these types of consortia before.

*During a 5-day start-up workshop, all consortia members in the CEPHaS programme came together for face-to-face discussions to help develop their programme’s theory of change. Several of the organisations were new to consortium-based research and this process helped everyone to understand their contributions and to align everyone with the intended outcomes.*

Consortia often put a lot of effort into understanding their partner organisations’ needs, and strengths and weaknesses, and to support areas that organisations found challenging. This early identification of weaknesses—often in data management and financial management of research—enabled consortia to put extra support in place, usually from within the consortia themselves.

*To rationalise and harmonise the consortia’s and organisations’ systems while still enabling them to meet funders’ requirements, a data storage and management platform was set up by IMPALA and hosted by one partner organisation that had capacity to safely store and manage all research data collected by partners.*

Some consortia invited their member organisations to start up meetings to make decisions about whether to centralise the management of finances and research data in the lead organisations or to decentralise it to member organisations. This prompted organisations to reflect on their own research systems. These open discussions were important because some organisations welcomed centralised management of funds as an efficient way to procure research consumables and equipment, whereas others thought that centralisation showed a lack of trust in their organisations’ capabilities. Joint written agreements were developed between consortia and organisations concerning, for example, financial responsibilities, data management, authorship and intellectual property.

Opportunities for feedback about management of the consortia, and for constructive and safe discussions were important for enabling all those involved in the consortium to air their personal concerns (eg, on workloads or inequities) to the management team. These feedback and communication processes tended to be refined and adapted as the programme and the relationships across the consortium matured. In some cases, this ‘learning’ about how to do better in terms of research capacity strengthening and in responding to feedback was formalised as the responsibility of a dedicated team.

*A ‘learning team’ embedded across the ACBI programme fed back anonymised concerns from consortia to the programme management team. This resulted in senior administrators visiting selected African research organisations to understand their challenges and work with them to find solutions and to introduce more flexibility in how organisations could spend funds.*

Although initially such discussions could be difficult, ultimately, having clarity and formal agreements around roles and responsibilities, and constructive feedback channels to a responsive management team, strengthened organisations’ research systems and engendered trust and respect between the consortia, the organisations and programme managers.

*Open and anonymous channels were established in several programmes (ACBI, PIIVeC, IMPALA) to discuss potentially contentious issues such as finances (eg, amounts and timings of transfers), data stewardship, intellectual property and publications and to provide transparency and opportunities about learning from mistakes, which helped to build trust. On occasions, these discussions were only among LMIC partners, with anonymised summaries fed back to the consortium leadership team.*

Measuring changes in research capacity is challenging since these may only become sustainable several years after the grant has ended and cannot easily be attributed directly to an individual programme. However, consortia did provide examples of how they evidenced the outcomes of their capacity strengthening efforts, which included improvements in infrastructure, grant income and organisations’ systems and processes.

*Examples from the DELTAS programme include:*

- *Physical infrastructure at member organisations provided through consortia resources, including a Research and Training Block, trainee accommodation and increased internet bandwidth, continued to serve other research and training activities beyond the programme*.
- *A member organisation received grants awards from two major research funders after training received on grant acquisition and management as part of consortia activities*.
- *A member organisation adopted grant management processes learnt from a consortium and arranged visits to other member organisations in the consortia*.
- *A consortium tracked trained researchers’ progress for several years after graduation and demonstrated their research and management roles in their organisations and how they are contributing to their organisation’s research systems*.
- *Institutionalised grant management systems put in place to manage consortia activities were used to enhance management of other grants*.
- *Member organisations leveraged the influence of the consortium to persuade organisational leaders to take decisions on, or provide resources for, activities that promoted research including adopting a student supervision policy and support internet improvement project*.

## Recommendations

Research consortia can play a major role in strengthening the research systems and promoting conducive research cultures in their member organisations. Ultimately, this not only enhances the quality of all the organisations’ research, but it also ‘leads to increased organisational resilience’ and the consolidation of research capacity.^3^ Currently, there is still a low level of investment in organisations’ research systems, especially in human resources such as professional services and research support staff, and this often leads to insufficient project management capacity across LMIC partners, especially in project execution, procurement and monitoring and evaluation.^21^

Many of the consortium-related benefits for organisations’ research systems can be realised through strategic investment in individuals,^22^ because a conducive environment will attract and retain a high-calibre research workforce. Consortia leaders therefore need to know how to achieve the dual benefits of developing individuals and aligning the development of individuals to organisations’ needs. This means being inclusive by providing equitable opportunities, not just for academics, but for all those involved in the research process (eg, research professional staff including librarians, accountants, IT technicians and those responsible for research governance).^22^

Although all the consortia we examined had a mandate from the funders to strengthen research capacity, they were given little guidance by the funders about how this could be best achieved. Furthermore, consortia leaders were primarily selected for their research expertise rather than their knowledge of good practice in strengthening research systems and could have benefited from more guidance and resources at the start. Consortia can open up training opportunities for staff in their member organisations who are not directly supported by the consortium and they can facilitate cascading of new skills and knowledge.^22^ Funders should encourage and monitor these activities since they are valuable and cost-effective ways of enhancing organisations’ research capacity.^18^

This ethos of inclusivity should also extend to valuing the contributions of all disciplines represented across consortia organisations. Multidisciplinary research is essential to tackle complex health, climate and other priority issues, and member organisations’ research systems and cultures need to be able to integrate and equitably support multiple disciplines. Consortia provide a good platform for bringing together diverse expertise but need to negotiate different power relations across their member organisations.^23–26^ It takes considerable—and frequently underestimated—time and resources to break down barriers and to create trusting and respectful relationships among disciplines.^27 28^ Unless this process is managed well and early, disunity can build up^29^ and consortium members may become stressed and demotivated, which may jeopardise the functioning of the whole consortium.^18 30 31^

Empowering individuals with knowledge and skills in management and research leadership benefits the consortium and also contributes to strengthening member organisations’ research systems.^32^ Consortia can promote empowerment by involving members in the active running of the programme^18^ and by capacitating them to take on increasing responsibilities within the consortium and in their organisations over the consortium’s lifetime. This requires a focus, not only on technical training and formal processes, but also on ‘soft’ skills such as communication and being respectful and equitable.^18^ It also requires mechanisms that allow consortium members to safely provide feedback and air concerns to optimise the capacity strengthening gains.^18 22 33^

Participating in and managing programmes that have dual ‘research’ and ‘capacity strengthening’ goals take substantial time for consortia and their member organisations, which is often underestimated. They need to balance efficiency (eg, a centralised management structure) and effectiveness (eg, decentralisation with strengthening of processes in partner organisations) of programme delivery.^34–39^ These two concepts—efficiency and effectiveness—could be complementary if consortia receive clear guidance from funders about their commitment to improving organisations’ research systems.^40 41^ This commitment should be reflected in funders’ requirements at all stages of the research process, from designing schemes and commissioning projects, to monitoring and evaluation.^38^ Funders’ selection panels should have expertise in, and weight their scores appropriately towards, strategies for strengthening organisations’ research systems.^42^

The more engaged a partner organisation is in consortia management, leadership and decision-making, the greater the benefit to both consortium and partner, and the more research capacities and systems will be strengthened.^18^ However, there has been almost no research into how to do this.^17^ An evidence-informed framework based on a theory of change^43^ has recently been published to help consortia with decision-making so they can maximise research capacity gains and returns on investments.^18 44^ However, without more knowledge of what works in different contexts and what does not, many opportunities to strengthen organisations’ research environments may be overlooked and, therefore, not included in consortia’s budgets and activities. Generating such knowledge is difficult because gains in research capacity may not become apparent until a few years after the end of a project, and by then attribution of any changes to an individual project may be unclear. Despite this, if consortia could better demonstrate their role, even in part, in system changes, this would strengthen the evidence required to make a case for greater focus and investments.

To achieve a stepwise change in research systems strengthening, funders need to send a clear message to consortium leaders that an organisation’s research systems and processes, and their research outputs, should both be ‘excellent’ and complementary,^45^ and based on the principles of ethical and equitable partnerships.^33 46 47^ Without such explicit statements, consortium leaders will likely continue to prioritise research outputs over embedding sustainable improvements in their organisations’ research systems.^18^ Funders need to recognise that outcomes for system-level research capacity strengthening efforts are not always quantifiable or produced in the short term, and they should be open to and work towards developing a wide range of evaluation indicators for assessing consortia performance. Much more evidence is needed about how to evaluate the research capacity strengthening components of consortia-based programmes. This includes better, validated tools and indicators and evaluation processes that are constructive for funders and grantees, and not overly burdensome. This means that funders need to identify knowledge and practice gaps and commission research to fill these gaps.

Our recommendations (box 2) are based on real-life evidence from several large consortia-based research programmes and, if implemented, they could contribute substantially to improving the design and operation of consortia-based programmes so they can more effectively achieve their research capacity strengthening outcomes. The transferability of our recommendations is enhanced because they are based on evidence collected through a variety of research methods, from programmes covering diverse research topics and from the multiple perspectives of different members of the research workforce including researchers, research managers, institutional directors, research funders, laboratory technicians and programme evaluators. However, the majority of organisations involved were academic and based in sub-Saharan Africa so the applicability of our findings beyond these contexts may be limited. It is possible that there may have been a bias among participants to over-report positive examples and so we may have missed covering some of the less successful aspects in our recommendations. However, we sought to mitigate this by ensuring that participants all understood that our learning research team was independent of the grant managers and research funders, and that the purpose of our involvement was to use their (anonymised) contributions to make positive ongoing improvements to their programme.

Box 2 **Recommendations for research funders and consortium leaders about how consortia with a mandate to strengthen research capacity can contribute to reforming and strengthening organisations’ research systems and also contribute to future learning**

*For funders*

Improving research capacity within consortia’s lifetime

- Make it explicit in grant calls that consortia need to take a systems approach to strengthening research capacity (ie, beyond individuals) and that they need to provide evidence-based, systematic plans (including for strengthening organisational research systems) and indicate how progress will be tracked.
- Provide guidance and resources to successful applicants including the expected weighting of research capacity strengthening efforts compared with the primary research outputs.
- Ensure consortia have allowed enough time to achieve the dual goals of conducting research and improving research systems since they will need longer than programmes without this intensive capacity strengthening component.
- Ways of strengthening research capacity need to be very responsive to context so funders need to be flexible and broad-minded about the wide range of potential capacity changes when evaluating consortia. This could include achievements that are tangible and intangible, technical and managerial, quantifiable and unquantifiable, strategic and operational, programme oriented and organisation oriented, short and long term, and whether wholly or partially attributable to the programme.

Contributing to future learning

- Ensure applicants include ways of sharing and cascading knowledge and skills beyond the direct consortium beneficiaries—include this in the grant requirements and in the project evaluation.
- Consortia should embed a ‘learning and research’ team in their programme capable of conducting the type of predominantly qualitative research needed to facilitate and track improvements in organisations’ research systems—this should be reflected in the scheme notes and guidance for applicants.
- Create funding schemes for research on strengthening organisational research capacity, including ways to orientate consortia management processes towards this goal.

*For consortium leaders and members*

Individual level

- Make sure that training and development opportunities are provided equitably to all those involved in the consortium, not just researchers and academics.
- Consider providing training opportunities to organisations’ staff beyond those directly supported by the consortium, including staff in technical and professional services.
- Provide language training and translations for those who are not primarily English speakers (or who do not speak the primary language used by the consortium).
- Actively promote interactions across multiple disciplines. Allow enough time and budget to overcome disciplinary boundaries effectively and to generate mutual respect and understanding.

Organisational level

- Initiate early and individualised discussions with each member organisation, promote leadership by low/middle-income country partners and agree responsibilities for managing finances, data, intellectual property and publication authorship.^54^
- Identify strengths and weaknesses in member organisations’ systems and facilitate sharing of expertise across the consortium to address any gaps.
- Prospectively decide on and measure indicators that demonstrate strengthening of research systems in member organisations.
- Put safeguarding mechanisms in place, nominate safeguarding leads for each partner organisation, provide them with appropriate training and make sure everyone in the consortium knows that safeguarding is their responsibility.^19^ ^20^

(Inter)national level

- From the start, make sure the consortium’s research and capacity strengthening goals meet priority needs of partners and their countries, and that the synergies offered by consortia’s interorganisational collaborations are used to catalyse the uptake of research findings.
- Consider longer-term and system-level research gains when developing consortia goals and activities and allocating resources.
- Develop strategies to help organisations sustain productive research and mentoring collaborations and networks beyond the life of the consortium.

## Data Availability

Data are available upon reasonable request.
